# Editorial: Transcatheter Aortic Valve Implantation: All Transfemoral? Update on Vascular Acccess and Closure

**DOI:** 10.3389/fcvm.2022.907445

**Published:** 2022-05-05

**Authors:** Alfredo Giuseppe Cerillo, Andreas Voetsch, Jonathan Michel, Hendrik Ruge

**Affiliations:** ^1^Unit of Cardiovascular Surgery, Careggi University Hospital, Florence, Italy; ^2^Department of Cardiovascular and Endovascular Surgery, Paracelsus Medical University, Salzburg, Austria; ^3^Unit of Interventional Cardiology, University Hospital Zurich, Zurich, Switzerland; ^4^INSURE (Institute for Translational Cardiac Surgery), Department of Cardiovascular Surgery, German Heart Center, Technical University of Munich (TUM), Munich, Germany; ^5^German Heart Center Munich, Department of Cardiovascular Surgery, Technical University of Munich (TUM), Munich, Germany

**Keywords:** TAVI – transcatheter aortic valve implantation, transfemoral access, aortic stenosis, intravascular lithoplasty, transcaval access and closure, transsubclavian transcatheter aortic valve replacement, transapical access, transaortic access

## Tavi Related Vascular Complications: Do They Still Exist?

According to the Transcatheter Valve Therapy (TVT) Registry, transcatheter aortic valve implantation (TAVI) has become the predominant treatment for aortic valve stenosis. The volume of TAVI exceeded the number of isolated surgical aortic valve replacement (AVR) procedures in 2015, and that of all forms of AVR in 2018. In the USA, in 2019, a total of 77,991 TAVI and 57,626 AVR were performed (1).

Thanks to increasing operator and institutional experience, to the expansion of the treatment indication to intermediate and low-risk patient populations, and to the fast evolution of transcatheter technologies, the rate of TAVI-related major complications has consistently reduced over the previous decade (1). However, TAVI complications remain of concern, and have a significant influence on morbidity and mortality.

*VARC-3 major vascular access site complications*, defined as a vascular injury leading to death, life threatening of major bleeding, visceral ischemia or neurological impairment, represent a significant proportion of the TAVI-related complications, and constitute a significant burden for the patient and for health care facilities (2). According to the TVT Registry the rate of thirty-day major vascular access site complications has declined from 1.6% in 2013 to 1.3% in 2019, but due to the significant increase of the total number of procedures performed, the number of patients suffering from a major vascular complication has increased from 39 to 922 during the same period (1).

## Improved Planning, Better Devices, New Technologies: Toward the Universal Trans-Femoral (TF) Approach

**Figure 1:**
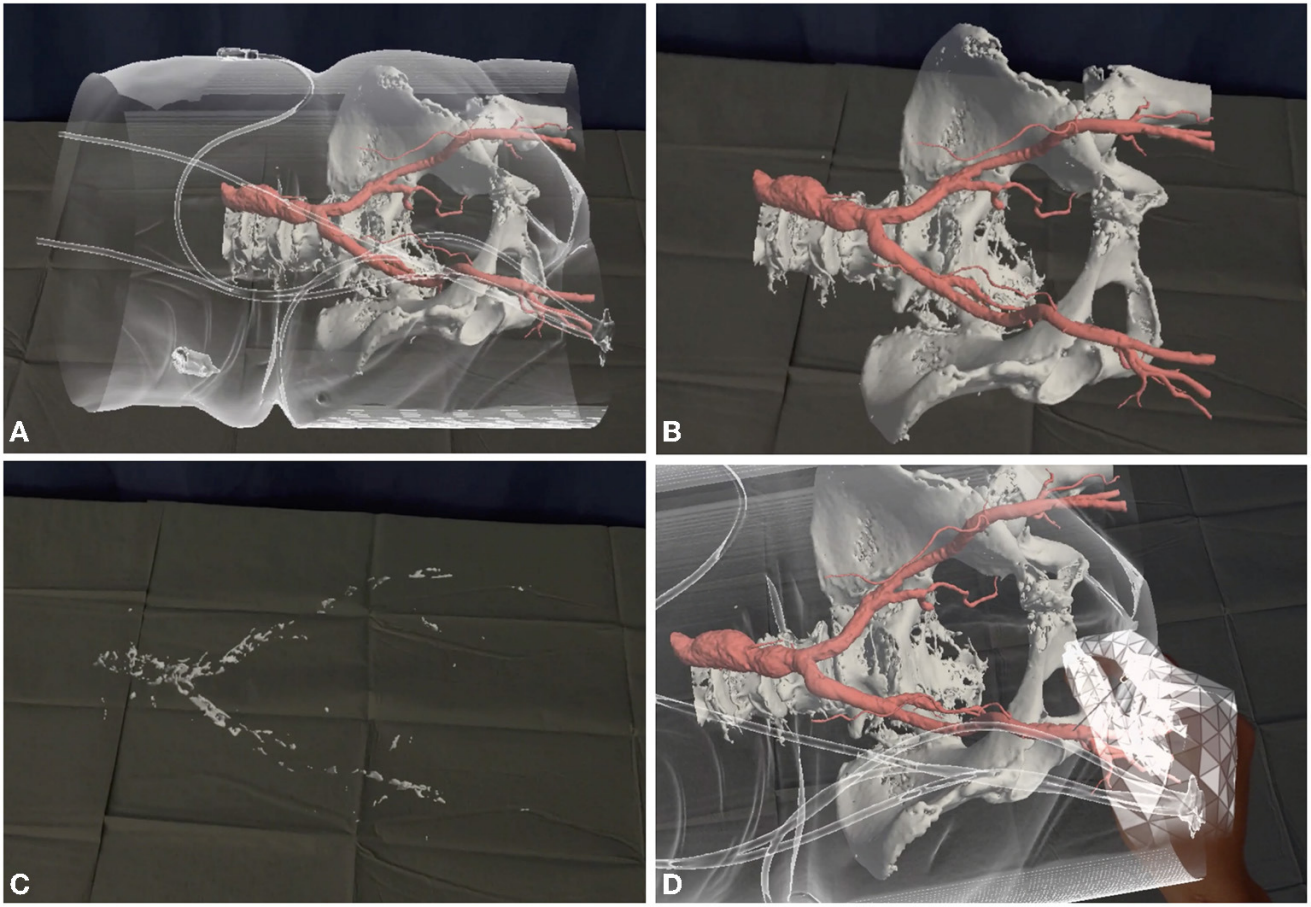
Augmented reality can simplify vascular access. The patients' CT scan is reconstructed with a proprietary software (Articor, Artiness SRL, Milano, Italy) to generate 3D holograms, that can be moved to the hybrid room and visualized with commercial holographic viewers (Hololens 2, Microsoft Inc, Redmonds, Washington, US). **(A)** The patients' arterial tree is seen through the transparent skin surface. **(B, C)** Different layers can be visualized or removed, to analyze different aspects of the vascular access (size, tortuosity, calcifications). **(D)** A dedicated tool can be used to simulate vascular access.

The progressive reduction of the rate of vascular complications is the result of improved preoperative assessment and improved technologies. Perrin et al. concluded in their comprehensive review that pre-procedural planning is the key for a successful TAVI program. Tridimensional curved multiplanar reconstruction of the CT images of the vascular path is a powerful tool to anticipate - and avoid - problems; echo-guided vessel puncture may be extremely helpful, and there is evidence that it can significantly reduce the rate of vascular complications (3); finally, the recent boost of virtual and augmented reality could further facilitate vascular access (Figure 1).

Beside improved planning, smaller delivery systems and improved vascular closure devices allow to safely navigate the transfemoral route in an increasing number of patients. While the suture-based Perclose ProGlide (Abbott cardiovascular, Plymouth, MN) remains the most widely used, newer vascular closure devices could improve the results in patients with complex access, as outlined in the articles by Schaefer et al. and Heitzinger et al.

In heavily-calcified access vessels, preoperative angioplasty can be used to navigate complex iliofemoral axes: as described by Thieme et al., most access site complications can be successfully treated with an interventional approach, and the same techniques can be used to pre-dilate stenotic vessels. More recently, balloon lithoplasty has proved to be a useful tool to cross calcific stenoses. In Florence, we've used this approach in several TF patients and in a trans-axillary procedure, that is described in detail here, while the article by Sawaya et al. offer an extremely detailed, step-by-step description of this technique, and report on an impressive series of 50 patients.

## Is There Still a Role For Alternative Access Routes?

As TAVI is becoming the standard treatment for calcific aortic stenosis, more and more low-risk patients are assigned to this procedure - many of them with “Less than perfect” vascular access. While skilled operators equipped with the latest technologies can perform complex TF-TAVI and succeed, a proportion of patients will still experience major vascular complications. The rate of complications will indubitably be related to the bravery of the operators: the more intrepid the physician, the higher the rate of vascular damages to be repaired. While some patients present with unfavorable vascular anatomy at every potential access route While for some patients there are no alternatives (or the alternatives are all really bad, and therefore require that is when brave and skilled operators is needed), in many other cases a safe alternative is available. Trans-axillary TAVI can be performed under loco-regional anesthesia (as in the case presented in this issue) and is a valuable option in many cases not eligible for TF-TAVR. Trans-carotid TAVI has been proven to be safe and effective, and can be also performed under loco-regional anesthesia, as outlined in the reviews by Stastny et al. and Perrin et al.

Surgical access TAVI is more traumatic and is associated with longer hospital stay and with a higher incidence of selected complications. However, as outlined in the review by Stastny et al., trans-aortic and trans-apical TAVI may be a valuable option for selected patients, and should be available – at least in high volume centers – to allow for a tailored, patient-centered approach.

Finally, the transcaval route – which is nicely illustrated here by Barbash et al. and by Perrin et al. - can be safely used in selected patients although, as outlined by Perrin, it requires some experience.

As TAVI is developing toward the predominant therapy for patients suffering from aortic valve stenosis, the procedure itself still presents challenges. With the expansion toward intermediate and low-risk patients, optimal procedural outcomes regarding stroke, valve hemodynamics, permanent pacemaker requirements, and vascular complications are mandatory. Currently, TAVI procedures are mostly restricted to high-volume centers. Their experience and access to the latest technologies and improvements allows optimal procedure planning including choice of vascular access to optimize patient outcome in TF-TAVI.

## Author Contributions

All authors listed have made a substantial, direct, and intellectual contribution to the work and approved it for publication.

## Conflict of Interest

The authors declare that the research was conducted in the absence of any commercial or financial relationships that could be construed as a potential conflict of interest.

## Publisher's Note

All claims expressed in this article are solely those of the authors and do not necessarily represent those of their affiliated organizations, or those of the publisher, the editors and the reviewers. Any product that may be evaluated in this article, or claim that may be made by its manufacturer, is not guaranteed or endorsed by the publisher.
